# Renal Function in Chinese HIV-Positive Individuals following Initiation of Antiretroviral Therapy

**DOI:** 10.1371/journal.pone.0135462

**Published:** 2015-08-28

**Authors:** Yan Zhao, Mingjie Zhang, Cynthia X. Shi, Yao Zhang, Weiping Cai, Qingxia Zhao, Yong Li, Huiqin Li, Xia Liu, Limeng Chen, Ye Ma, Fujie Zhang, Zhongfu Liu, Zunyou Wu

**Affiliations:** 1 National Center for AIDS/STD Control and Prevention, Chinese Center for Disease Control and Prevention, Beijing, China; 2 Guangzhou 8^th^ Hospital, Guangdong, China; 3 Zhengzhou 6^th^ Hospital, Henan, China; 4 Longtan Hospital, Guangxi, China; 5 Center for AIDS Care, Yunnan, China; 6 Peking Union Medical College Hospital, Chinese Academy of Medical Sciences, Beijing, China; University of Sao Paulo Medical School, BRAZIL

## Abstract

**Aim:**

To identify the prevalence and predictors of abnormal renal function among HIV-positive Chinese patients prior to antiretroviral therapy (ART) initiation and to evaluate subsequent changes in renal function after ART exposure.

**Methods:**

We conducted a nationwide cohort study of subjects who enrolled in the national Chinese ART program from January 1, 2012 to December 31, 2012. We estimated the glomerular filtration rate (eGFR) of subjects prior to and after initiating ART. Risk factors for abnormal renal function, as defined by eGFR <60 ml/min/1.73m^2^, at baseline and follow-up were assessed by logistic regression and Cox proportional hazards regression models, respectively.

**Results:**

Among 41,862 subjects, at ART baseline, 3.3% had a baseline eGFR <60 ml/min/1.73m^2^ and 24.2% had eGFR = 60–90 ml/min/1.73m^2^. Adjusted baseline risk factors for baseline eGFR <60 ml/min/1.73m^2^ were older age (Adjusted odds ratio [AOR] = 5.19, 95% confidence interval [CI]: 4.52–5.67), female (AOR = 1.68, 95% CI: 1.47–1.93), hemoglobin <120g/L (AOR = 1.68, 95% CI: 1.47–1.93), blood glucose >6.1 mmol/L (AOR = 1.46, 95% CI: 1.25–1.72), and hepatitis C co-infection (AOR = 1.36, 95% CI: 1.06–1.73). Among subjects with baseline eGFR >90 ml/min/1.73m^2^, the incidence of the eGFR falling to <60 ml/min/1.73m^2^ was 0.92/100 person-years after a median of 15.0 months of ART. Being on a tenofovir with lopinavir/ritonavir regimen (Adjusted hazard ratio [AHR] = 3.02, 95% CI: 1.96–4.66) and having an unsuppressed viral load (AHR = 2.70, 95% CI: 1.80–4.03) were independent predictors for eGFR <60 ml/min/1.73m^2^ after ART initiation as well as older age, female, and hemoglobin <120 g/L.

**Conclusion:**

A high proportion of HIV-positive subjects in China presented with abnormal renal function prior to ART initiation. But the incidence of the eGFR decrease after ART was low. Patient renal function should be regularly monitored by eGFR before initiating and during ART.

## Introduction

Compared to the general population, HIV-positive patients experience a higher risk of renal function impairment, chronic kidney disease [1], end-stage renal disease, and renal death [2, 3]. Kidney disease predicts accelerated HIV disease progression and increased mortality [4]. The reported prevalence of CKD among the HIV-positive population varies worldwide [4], ranging from 2–48.5% of HIV-positive patients [5, 6]. The prevalence of CKD among HIV-positive patients has been estimated as 3.5–18% in 31 European countries, 1.1–5.6% in Brazil, 27% in India, and 6.0–48.5% in sub-Saharan Africa [7]. Among patients who are not on antiretroviral therapy (ART), HIV-associated nephropathy [8] and HIV immune complex-mediated kidney disease are major concerns. Current guidelines recommend that all HIV-positive individuals undergo screening for kidney function by calculating the estimated glomerular filtration rate (eGFR) [9].

Known risk factors for CKD among HIV-positive patients are black race [10], older age, CD4 count <200 cells/mm^3^, HIV RNA levels >4,000 copies/ml, family history of renal disease, clinical progression to AIDS, diabetes mellitus, hypertension, and co-infection with hepatitis B (HBV) or Hepatitis C (HCV) [4]. While sustained ART is associated with lower incidence of renal disease and significant improvement in kidney function [11–13], there is continued debate about the impact of nephrotoxic antiretroviral agents [14]. Recent research has focused on tenofovir disoproxil fumarate (TDF), ritonavir-boosted atazanavir (ATV/r), and lopinavir/ritonavir (LPV/r), which have been reported to be independent predictors of chronic renal impairment in HIV-positive patients without previous renal disease [2].

In China, several small cohort studies have reported high CKD incidence rates in HIV-positive ART-naive patients, but no nationwide study has previously addressed renal function at baseline among HIV-positive patients [15]. We aim to assess the prevalence and associated risk factors of abnormal renal function at baseline and of changes in renal function over the first 24 months following ART initiation.

## Methods

### Study population

**Fig 1:**
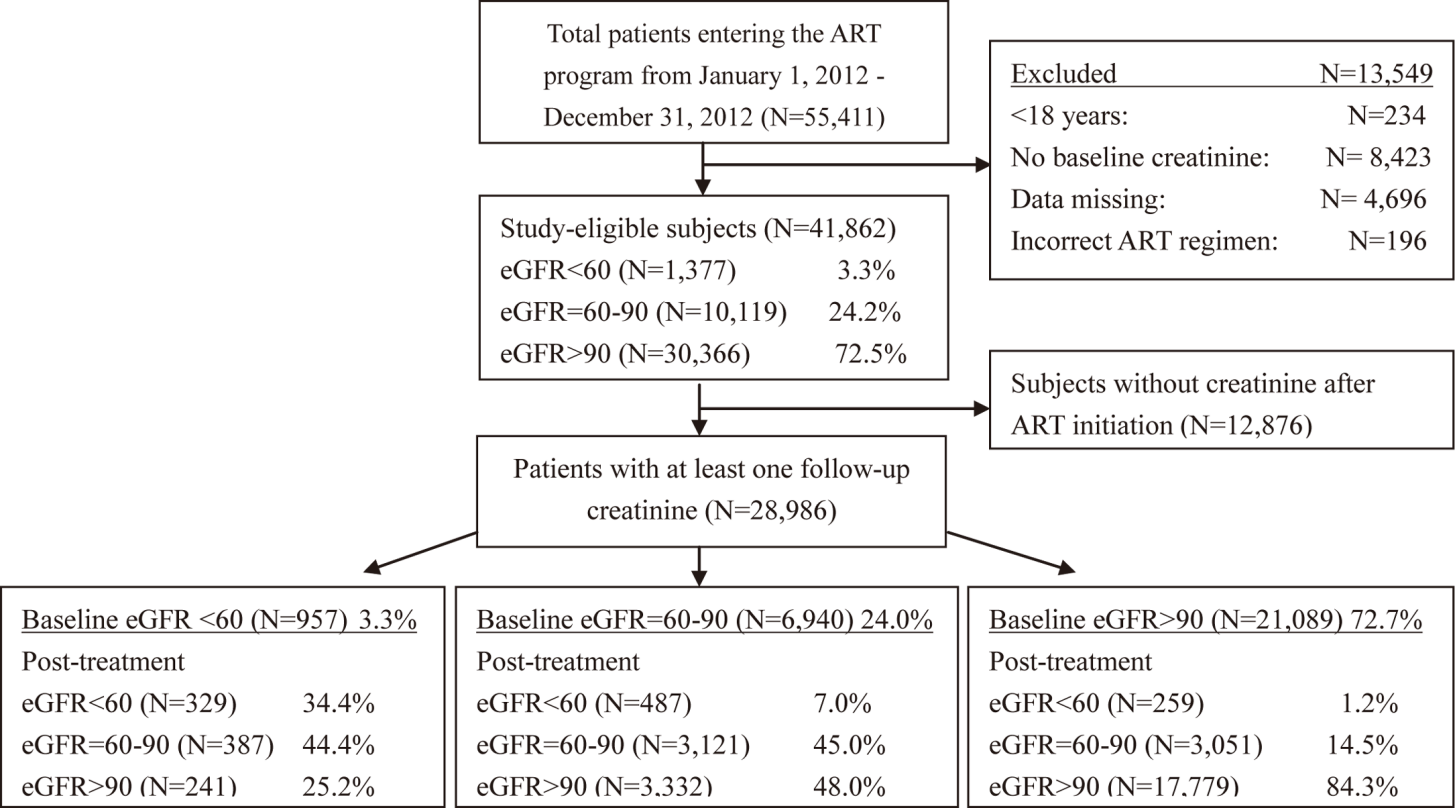
Development of the study cohort.

This study retrospectively assessed a nationwide cohort of Chinese HIV-positive subjects who initiated ART from January 1, 2012 to December 31, 2012 (Fig 1). Each case was observed for 24 months after ART initiation. The study follow-up endpoint was December 31, 2014. Eligible subjects had at least one serum creatinine measurement prior to ART, were >18 years old, had documented age, sex, and weight at baseline, and had a recorded ART regimen. Subjects without baseline creatinine measurements were excluded from the final study analysis.

### Data collection

Data were abstracted from the Chinese National Free ART Program (NFATP) database, a real-time web-based ART data system covering all ART-providing clinics nationwide. This database contains demographic data and detailed information on ongoing clinical management, including ART treatment regimens [4]. Data are entered by clinicians based on nationally standardized clinical report forms, which include sex, age, ethnicity, route of transmission, weight, World Health Organization (WHO) HIV/AIDS clinical stage, hemoglobin test results, HBV antigen test results, HCV antibody test results, CD4 counts, ART regimen and doses, viral load, drug side effects, and serum creatinine. Data on diabetes, hypertension, family history of renal disease, and urine analysis results are not available through the NFATP database. Body mass index (BMI) scores were generated by the formula (weight in kilograms)/(height in meters)^2^. Records of HIV treatment regimens were uploaded upon ART initiation and updated at each follow-up. Patients are asked to return for routine clinical follow-up visits every 3 months. CD4 testing was conducted every 6 months, and viral load testing was provided every 12 months following ART initiation.

In China, HIV-positive individuals are eligible for ART initiation when they meet the national treatment criteria: CD4 <350 cells/mm^3^ for all HIV-positive patients, CD4 <500 cells/mm^3^ among patients >65 years, and at all CD4 levels for serodiscordant couples [16]. The available ART drugs are TDF, zidovudine (AZT), stavudine (D4T), lamivudine (3TC), nevirapine (NVP)/efavirenz (EFV), and LPV/r. TDF has been a first line regimen since 2012. LPV/r is used as an alternate regimen due to potential toxicities with non-nucleoside reverse transcriptase (NNRTI). For this study, ART regimens were categorized into four types according to whether they included a NNRTI, LPV/r, or TDF.

### eGFR calculation

The Modification of Diet and in Renal Disease (MDRD) equation was used to generate the patient eGFR [17]. The MDRD equation is: eGFR = 186 × serum creatinine (mg/dl) − 1.154 × [age (years)] − 0.203 × [0.742 if female] × [1.212 if black]. Renal function was evaluated according to the staging system of the Kidney Disease Outcomes Quality Initiative (K/DOQI) [18]. Baseline eGFR results must have been conducted within 4 weeks prior to ART initiation. Because most subjects only had one baseline eGFR value at the time of ART initiation and we did not have data on kidney disease history and other clinical examinations, we did not categorize subjects as having CKD. Subjects were categorized as by their eGFR values <60, 60–90, or >90 ml/min/1.73m^2^, according to the defined eGFR threshold for CKD. After ART initiation, if subjects had multiple documented eGFR results, the last test result within the observational period was used.

### Statistical analysis

Categorical variables are presented as numbers and percentages, and continuous variables are described using medians and interquartile ranges (IQR). The study follow-up for each subject was conducted from the ART initiation to 24 months after ART initiation. Subjects who had valid creatinine measurements before and after ART initiation within the defined periods were assessed for changes in eGFR. CD4 count was classified into 3 groups: <200, 200–350, and >350cells/mm^3^, and viral load suppression was defined as <400 copies/ml.

Risk factors for baseline eGFR <60 ml/min/1.73m^2^ were assessed by logistic regression models. For subjects who had eGFR >90 ml/min/1.73m^2^ prior to initiating ART, we calculated the incidence rate of dropping to eGFR <60 ml/min/1.73m^2^. The total observed time was calculated in person-years from ART initiation to the last creatinine measurement within the defined study period. Changes from eGFR >90 ml/min/1.73m^2^ at ART baseline to eGFR <60 ml/min/1.73m^2^ after ART were evaluated by Cox proportional hazards regression analysis. Statistically significant variables in univariate analysis were included in the multivariable model.

All p-values were 2-sided, and p<0.05 was considered statistically significant. Statistical analyses were performed using SAS software (Version 9.1.3, SAS Institute Inc., USA).

### Ethical approval

This study was reviewed and approved by the Institutional Review Board of National Center for AIDS/STD Control and Prevention. All data included in this study were extracted from the NFATP database. All participants provided signed routine informed consent when enrolling in the NFATP, and no further informed consent was required. Patient information was de-identified for data analysis.

## Results

### Study Cohort at Baseline

A total of 55,411 patients entered the NFATP from January 1, 2012 to December 31, 2012. Approximately 24% of patients (N = 13549) were excluded: 234 <18 years old, 8,423 did not have at least one recorded baseline creatinine result before ART initiation, 4,696 did not have either age, sex, or weight recorded at baseline, and 196 patients had missing or incorrectly documented ART regimens. We assessed the baseline homogeneity between subjects who were included and excluded from the baseline and follow-up analyses, and we did not find any significant differences in baseline characteristics (data not shown). There were 41,862 patients with a measured baseline eGFR prior to ART initiation who comprised the study cohort. At baseline, 3.3% (N = 1,377) of patients had eGFR <60 ml/min/1.73m^2^, 24.2% (N = 10,119) of patients had eGFR = 60–90 ml/min/1.73m^2^, and 72.5% (N = 30,366) had eGFR >90 ml/min/1.73m^2^ (Fig 1)

The median age of the study cohort was 38 years (IQR: 30–48), and 22.1% of subjects were over 50 years of age. Males comprised 68.5% of the study cohort. Body mass index (BMI) scores were available for 33925 subjects; the median BMI was 20.8 (IQR: 19.1–22.9), and the majority (66.8%) of subjects had BMI scores within the normal range of 18.5 to 24.0. Seropositivity for HBV or HCV co-infection had a prevalence of 7.8% and 11.0%, respectively. Of the 40,503 subjects with available CD4 count results at baseline, the median was 220 cells/mm^3^ (IQR: 105–380), and 86.2% had CD4 <350 cells/mm^3^. Most subjects (65.5%) were classified as WHO clinical stage 1 or 2, and 34.5% were diagnosed as stage 3 or 4. The median hemoglobin level was 134 g/L (IQR: 117–148), and 27.6% had hemoglobin <120 g/L. About 15.9% of the cohort had been treated with trimethoprim/sulfamethoxazole (TMP-SMZ). The median blood glucose level was 5.1 mmol/L, with 11.8% of subjects with results >6.1 mmol/L(Table 1).

**Table 1:** Predictors of the baseline eGFR<60 ml/min/1.73m^2^.

Variable	Total N(%)	eGFR<60 ml/min/1.73m^2^ N(%)	Unadjusted OR (95% CI)	P-Value	Adjusted OR (95% CI)	P-Value
Overall	41,862	1,377(3.3%)				
Age (years)						
Median (IQR)	38 (30–48)	54.0 (41.0–64.0)				
<50	32,478 (77.6%)	594(43.1%)	1.00		1.00	
≥50	9,256(22.1%)	783(56.9%)	4.96 (4.45–5.53)	<0.001	5.19 (4.52–5.67)	<0.001
Missing	128(0.3%)					
Sex						
Male	28,685(68.5%)	770(55.9%)	1.00		1.00	
Female	13,177(31.5%)	607(44.1%)	1.75 (1.57–1.95)	<0.001	1.68 (1.47–1.93)	<0.001
BMI						
Median (IQR)	20.8 (19.1–22.9)	20.8 (19.0–22.9)				
<25	30,852(73.7%)	1,076(78.1%)	1.00			
≥25	3,037(7.3%)	114(8.3%)	1.07 (0.88–1.30)	0.528		
Missing	7,937(19.0%)	187(13.6%)				
Baseline CD4 (cells/mm^3^)						
Median (IQR)	220 (105–308)	197.0 (88.0–290.0)				
<200	18,201 (43.5%)	664(48.2%)	1.46 (1.22–1.77)	<0.001	1.05 (0.82–1.34)	0.780
200–349	16,721(39.9%)	508 (36.9%)	1.22 (1.01–1.47)	0.956	1.15 (0.92–1.46)	0.130
≥350	5,581 (13.3%)	140 (10.2%)	1.00		1.00	
Missing	1,359(3.2%)	65(4.7%)				
WHO Clinical Stage						
1–2	27,424(65.5%)	841 (61.1%)	1.00		1.00	
3–4	14,437(34.5%)	536(38.9%)	1.22 (1.09–1.36)	<0.001	1.02 (0.88–1.18)	0.831
Missing	1(0.0%)					
Hemoglobin						
Median (IQR) (g/L)	134 (117–148)	121.0 (103.0–136.0)				
<120	11,549 (27.6%)	649 (47.1%)	2.44 (2.19–2.72)	<0.001	1.84 (1.59–2.13)	<0.001
≥120	29,716 (71.0%)	708(51.4%)	1.00		1.00	
Missing	597(1.4%)	20(1.5%)				
TMP-SMZ						
Yes	6,653(15.9%)	247 (17.9%)	1.16 (1.01–1.34)	0.035	0.98 (0.81–1.18)	0.840
No	35,209(84.1%)	1,130 (82.1%)	1.00		1.00	
Blood glucose						
Median (IQR) (mmol/L)	5.1 (4.6–5.7)	5.2 (4.7–6.0)				
<6.1	31,064 (74.2%)	915 (66.5%)	1.00		1.00	
≥6.1	4,946(11.8%)	251(18.2%)	1.76 (1.53–2.03)	<0.001	1.46 (1.25–1.72)	<0.001
Missing	5,852(14.0%)	211(15.3%)				
Hepatitis B infection						
Yes	3,251(7.8%)	102(7.4%)	0.95 (0.77–1.16)	0.613		
No	38,611(92.2%)	1,275(92.6%)	1.00			
Hepatitis C infection						
Yes	4,613(11.0%)	109(7.9%)	0.69 (0.56–0.84)	<0.001	1.36 (1.06–1.73)	0.014
No	37,249(89.0%)	1,268(92.1%)	1.00		1.00	

eGFR: estimated glomerular filtration rate, OR: odds ratio, CI: confidence interval, IQR: interquartile range, TMP-SMZ: trimethoprim/sulfamethoxazole

Adjusted ORs were calculated through multivariable analysis using a logistic regression model.

### Risk factors for baseline renal impairment

In this study cohort (Table 1), the significantly associated baseline risk factors for eGFR <60 ml/min/1.73m^2^ were age ≥50 years (adjusted odds ratio [OR] = 5.19, 95% confidence interval [CI]: 4.52–5.67), female (adjusted OR = 1.68, 95% CI: 1.47–1.93), high glucose levels (adjusted OR = 1.46, 95% CI: 1.25–1.72), low hemoglobin <120 g/L (adjusted OR = 1.84, 95% CI: 1.59–2.13), and infection with HCV (adjusted OR = 1.36, 95% CI: 1.06–1.73).

### Following ART initiation

The 28,986 subjects who had at least one creatinine value after initiating ART are described in Fig 1, stratified by baseline eGFR. The 957 subjects who had a baseline eGFR <60 ml/min/1.73m^2^ were observed for a median of 15.0 months (IQR: 11.0–21.0). At follow-up, 34.4% still had eGFR <60 ml/min/1.73m^2^, 241 subjects (25.2%) recovered to eGFR >90 ml/min/1.73m^2^, and an additional 387 subjects (44.4%) experienced eGFR = 60–90 ml/min/1.73m^2^. and the median value of change in increased eGFR was an increase of 20.2 (IQR: 12.7, 29.0) ml/min/1.73m^2^.

For the 6,940 subjects who had eGFR 60–90ml/min/1.73m^2^ at baseline, 7.0% experienced a decreased in eGFR to <60 ml/min/1.73m^2^, 45.0% remained at eGFR = 60–90 ml/min/1.73m^2^, and 48.0% improved to eGFR >90 ml/min/1.73m^2^.

The 21,089 subjects who had a baseline eGFR >90 ml/min/1.73m^2^ were observed for a median of 15.0 months (IQR: 11.0–21.0) for a total of 28,062 person-years. At follow-up, 1.2% (259 cases) had eGFR <60 ml/min/1.73m^2^, 14.5% experienced eGFR = 60–90 ml/min/1.73m^2^, and 84.3% stayed at eGFR >90 ml/min/1.73m^2^. Among the subjects who started with a normal eGFR >90 ml/min/1.73m^2^ at baseline, the incidence of having an abnormal eGFR <60 ml/min/1.73m^2^ was 0.92/100 person-years. The incidence of GFR <60 ml/min/1.73m^2^ over the duration of ART was 3.05, 1.02, 0.80, and 0.68 /100 person-years at <6, 6–11,12–17, and 18–24 months.

### Risk factors associated with eGFR changes after ART initiation

Among subjects with normal baseline eGFR >90 ml/min/1.73m^2^, a regimen of TDF with LPV/r was significantly associated with an abnormal follow-up eGFR <60 ml/min/1.73m^2^ (adjusted hazard ratio [HR] = 3.02, 95% CI: 1.96–4.66), compared with other regimens. Unsuppressed viral load was also associated with eGFR <60 ml/min/1.73m^2^ (adjusted HR = 2.70, 95% CI: 1.80–4.03). Consistent with the baseline eGFR analyses, other risk factors for low eGFR after ART initiation included age ≥50 years (adjusted HR = 3.88, CI: 2.79–5.38), female sex (adjusted HR = 1.56, 95% CI: 1.12–2.18), and low hemoglobin <120g/l (adjusted HR = 2.12, 95% CI: 1.49–3.01). All data was showed in Table 2.

**Table 2:** Cox proportional hazards model for the development of eGFR <60 ml/min/1.73m^2^ after ART initiation among patients with baseline eGFR >90 ml/min/1.73m^2^.

Variable	Total (N)	Incidence of eGFR<60 (l/min/1.73m^2^)	Unadjusted HR (95% CI)	P value	Adjusted HR (95% CI)	P value
Overall						
Age (years)	21,089					
<50	17,646	0.68	1.00		1.00	
≥50	3,443	2.13	3.11 (2.42–4.00)	<0.001	3.88 (2.79–5.38)	<0.001
Sex						
Male	14,551	0.75	1.00		1.00	
Female	6,538	1.29	1.71 (1.34–2.18)	< .0001	1.56 (1.12–2.18)	0.009
BMI						
<25	15,531	1.01	1.00			
≥25	1,533	0.87	0.84 (0.52–1.36)	0.472		
Missing	4,025					
Baseline CD4						
<200	9,358	1.17	1.79 (1.17–2.73)	0.007	1.79 (0.93–3.42)	0.081
200–350	8,239	0.76	1.19 (0.76–1.86)	0.448	1.84 (0.98–3.46)	0.059
>350	2,947	0.63	1.00		1.00	
Missing	545					
WHO Clinical Stage						
1–2	13,485	0.81	1.00			
3–4	7,603	1.11	1.24 (0.97–1.59)	0.084		
Missing	1					
Hemoglobin (g/L)						
<120	5,734	1.81	3.03 (2.37–3.87)	<0.001	2.12 (1.49–3.01)	<0.001
≥120	15141	0.59	1.00		1.00	
Missing	214					
TMP-SMZ						
Yes	3,429	1.33	1.52 (1.14–2.09)	0.005	1.03 (0.67–1.58)	0.884
No	17,660	0.84	1.00		1.00	
Blood Glucose (mmol/L)						
<6.1	16,252	0.81	1.00			
≥6.1	2,325	1.05	1.25 (0.86–1.81)	0.242		
Missing	2,512					
Hepatitis B infection						
Yes	1,729	1.02	1.12 (0.74–1.70)	0.603		
No	19,360	0.91	1.00			
Hepatitis C infection						
Yes	2,341	1.02	1.18 (0.80–1.72)	0.407		
No	18,748	0.91	1.00			
ART Regimen						
TDF/NNRTI	4,335	1.06	1.38 (1.03–1.85)	0.032	1.25 (0.84–1.85)	0.273
TDF/LPV/r	639	2.95	3.87 (2.54–5.90)	<0.001	2.85 (1.57–5.18)	<0.001
Other/NNRTI	15,578	0.79	1.00		1.00	
Other/LPV/r	537	1.07	1.34 (0.66–2.72)	0.419	1.74 (0.84–3.62)	0.140
Viral load (copies/ml)						
<400	12,532	0.60	1		1	
≥ 400	1,339	1.51	2.67 (1.81–3.94)	<0.001	2.70 (1.80–4.03)	<0.001
Missing	7,218					

eGFR: estimated glomerular filtration rate, HR: hazard ratios, CI: confidence interval, TMP-SMZ: trimethoprim/sulfamethoxazole, ART: antiretroviral therapy, TDF: tenofovir disoproxil fumarate, NNRTI: non-nucleoside reverse transciptase, LPV/r: lopinavir/ritonavir.

Adjusted HRs were calculated using multivariable Cox models.

## Discussion

This is the first large-scale study in China to evaluate renal function among HIV-positive individuals before and after ART initiation. We observed a high proportion of subjects with abnormal eGFR before ART initiation. In our study cohort, an eGFR <60 ml/min/1.73m^2^ at baseline was strongly associated with older age, female sex, low hemoglobin, abnormal blood glucose levels, and co-infection with HCV. Among subjects with normal baseline eGFR >90 ml/min/1.73m^2^, we found that older age, female sex, low hemoglobin, and unsuppressed HIV viral load were independent predictors of a decrease in eGFR to <60 ml/min/1.73m^2^ after initiating ART. Compared with other combinations, TDF with LPV/r regimens were associated with eGFR <60 ml/min/1.73m^2^.

### Monitoring

A nationally representative cross-sectional study in China found the prevalence of eGFR <60 ml/min/1.73m^2^ was 1.7% (95% CI: 1.5–1.9%) in the general adult population [19]. Among HIV-positive subjects, we found a prevalence of 3.3%, which nearly twice as high as the national prevalence. In resource-limited settings, monitoring of renal function is often insufficient due to limited medical resources and insufficient awareness of the need for screening. Due to the high proportion of abnormal eGFR observed at the study baseline, we recommend that all HIV patients should be assessed for kidney function at time of diagnosis and ART initiation. Urinalysis testing and serum creatinine can detect proteinuria and abnormal renal function. In particular, monitoring of renal function is critically important in the management of HIV care for older patients and preparation for ART regimens [12, 20].

### Risk factors for renal impairment

ART is associated with an estimated 60% risk reduction for HIV-associated nephropathy [21, 22]. Emerging research has investigated the role of ART in improving renal function among the HIV-positive population, which is due in part to the sustained suppression of viral replication [23]. Our study findings are in line with this past research. We found significant improvement in eGFR after ART initiation among subjects with low eGFR <60 ml/min/1.73m^2^ at baseline and that unsuppressed viral load was associated with the deterioration of eGFR.

In our cohort, we observed a higher risk of impaired eGFR in association with regimens containing both TDF and LPV/r. Several ART drugs are thought to be associated with renal impairment. Two recent meta-analyses of TDF and renal safety have reported that TDF had a statistically significant association with impaired renal function compared to other regimens [24, 25]. Regimens containing TDF (especially regimens containing both TDF and either atazanavir, indinavir, or a ritonavir-boosted protease inhibitor such as LPV/r) can reduce eGFR, suggesting additive toxicity or interactions between drugs [26, 27]. The D:A:D study cohort found that initial ART regimens with tenofovir, a ritonavir-boosted atazanavir or lopinavir were independently associated with significantly higher risk of renal impairment compared to regimens containing any non-tenofovir, non ritonavir-boosted atazanavir or lopinavir [2]. It should be noted that overall CKD rates for the D:A:D study was comparatively low (0.6%) over the median 4-year follow-up duration (incidence of 1.33 cases/1000 person-years) [2]. The incidence rates of ART-associated renal impairment decreased after discontinuation of ART. Another study of long-term ART found that TDF-based regimens are associated with mild-to-moderate nephrotoxicity with significantly greater than abacavir regimens [27].

Most studies indicated that low CD4 count was an independent predictor for renal impairment (2, 7, 11). In our study, CD4<200 cells/mm^3^ was a significant risk factor for renal impairment in the univariate analysis. After adjusting for other confounders, there was still a slight increase in risk in CD4<200 cells/mm^3^ group compared to the higher CD4 group, although the effect was not significant.

Lower hemoglobin levels also strongly predicted lower eGFR at follow-up. Anemia is a common complication of CKD, renal function should be monitored for HIV-positive subjects with anemia[28].

### Improving Care

Guidelines for the management of HIV-positive patients recommend annual screening by urinalysis for proteinuria and eGFR or creatinine clearance for high-risk patients (persons of African descent, CD4 <200, diabetes, hypertension, or HCV) and prior to the initiation of ART [9]. The monitoring of creatinine levels was insufficient over the enrollment and follow-up of our study cohort. Training for Chinese HIV care providers on renal function screening and monitoring should be strengthened. We also recommend that nephrologists should be better integrated into HIV care teams for patients with renal disease. The functions of nephrologists in HIV care settings include diagnosing kidney disease, distinguishing ART-induced kidney injury from kidney disease, and recommending appropriate renal treatment [4]. Dose-adjusted ART regimens are essential for the optimal management of patients who have had prior renal disease complications.

### Limitations

First, there are different biomarkers available for monitoring renal function [29], and the available eGFR equations are biased for HIV-positive individuals [4] and for Chinese patients with CKD [30]. MDRD equation is an international screening tool for monitoring renal function. In a study among Chinese CKD patient population without HIV, the MDRD equation produced underestimated eGFR rates for patients with near-normal renal function as well as overestimated rates for patients with in advanced renal failure [31]. MDRD equations may need to be modified for use in HIV-positive Chinese patients.

Second, this observational study was conducted among subjects in routine HIV care, and thus, their initial ART regimens were not randomly assigned. There is potential for confounding by indication for differences in baseline characteristics among patients receiving different initial ART regimens. Although we used multivariate statistical models to control for confounding by indication and potential bias, there may remain residual confounding from unmeasured factors.

Third, this cohort is drawn from the currently enrolled NFATP patient population. If patients with severe kidney damage were not on ART, they would not have been included in this cohort, leading to a potential underestimation of the renal impairment prevalence. Additionally, some subjects were excluded from the baseline and follow-up analyses due to missing data, although we found no significant differences in baseline characteristics between included and excluded subjects.

Finally, our study was limited to a short observational period. Previous studies of HIV-positive patients with renal impairment has found that longer exposure to tenofovir was independently associated with greater eGFR decline [32]. Future research is needed to examine the clinical impact of long-term ART exposure on renal function in HIV-positive patients. Some clinical indicators (e.g. proteinuria and other potentially nephrotoxic drugs) were not available in our dataset, which may be a cause of bias.

## Conclusion

In our study cohort, we found a high level of abnormal renal function among HIV-positive subjects prior to ART initiation. But the incidence of the eGFR decrease after ART was low. ART regimens containing TDF and LPV/r were associated with a decrease in renal function as well as being older than 50 years, female, and having low hemoglobin levels. We recommend regular monitoring of eGFR for HIV-positive patients before and during ART with subsequent adjustments of drug regimens and dosages.
